# Both natural selection and isolation by distance explain phenotypic divergence in bill size and body mass between South Australian little penguin colonies

**DOI:** 10.1002/ece3.2516

**Published:** 2016-10-11

**Authors:** Diane Colombelli‐Négrel

**Affiliations:** ^1^ School of Biological SciencesFlinders UniversityAdelaideAustralia

**Keywords:** adaptation, conservation, natural selection, seabirds

## Abstract

Morphological variation between populations of the same species can arise as a response to genetic variation, local environmental conditions, or a combination of both. In this study, I examined small‐scale geographic variation in bill size and body mass in little penguins (*Eudyptula minor*) across five breeding colonies in South Australia separated by <150 km. To help understand patterns driving the differences, I investigated these variations in relation to environmental parameters (air temperature, sea surface temperature, and water depth) and geographic distances between the colonies. I found substantial morphological variation among the colonies for body mass and bill measurements (except bill length). Colonies further located from each other showed greater morphological divergence overall than adjacent colonies. In addition, phenotypic traits were somewhat correlated to environmental parameters. Birds at colonies surrounded by hotter sea surface temperatures were heavier with longer and larger bills. Birds with larger and longer bills were also found at colonies surrounded by shallower waters. Overall, the results suggest that both environmental factors (natural selection) and interpopulation distances (isolation by distance) are causes of phenotypic differentiation between South Australian little penguin colonies.

## 1 Introduction

Understanding mechanisms of population divergence is important for both evolutionary biologists and conservationists. Under allopatric model of speciation, variation between populations is often the first step toward reproductive isolation, which in turn can lead to speciation (e.g., Coyne & Orr, 2004; Mayr, 1963). Divergent populations may also represent significant evolutionary units (i.e., populations that are considered distinctive for conservation purposes; see Crandall, Bininda‐Emonds, Mace, & Wayne, 2000). Therefore, documenting population differentiation can be critical for implementing effective conservation measures to ensure that distinct morphological, behavioral, or ecological traits are preserved (see Lesica & Allendorf, 1995; Moritz, 1994).

In birds, divergence in morphology is theoretically governed by genetic factors (reviewed in Merilä & Sheldon, 2001) or arises as a response to local environment (e.g., Grant & Grant, 1989, 2006; Schluter, 2001). Within species, greater morphological variation is expected between populations that are geographically more distant and more likely to be displayed in species where populations are not connected by gene flow (Mayr & Diamond, 2001). Morphological variation across different geographic locations may also evolve as a result of local adaptation to environmental conditions, food availability, or interspecific competition (Coyne & Orr, 2004; Grant & Grant, 1989; Schluter, 2001), and gradual changes along environmental gradients generally suggest local adaptation to environmental conditions (Endler, 1977). Studies on avian bill variation in the Darwin's finches provide some of the strongest evidence for local adaptation in response to changes in dietary food (Boag & Grant, 1981; Lack, 1947) and ecological competition (Grant & Grant, 2006). For example, during drought conditions on Daphne Island, larger medium ground finches (*Geospiza fortis*) with larger bills showed higher survival because of their superior ability to crush the hard seeds (Boag & Grant, 1981). Studies in endotherms have also shown that individuals living in colder climates are generally larger in size or body mass, which would allow them to better preserve heat (Bergmann, 1848; Blackburn, Gaston, & Loder, 1999; James, 1970) or store larger amounts of body reserves and thus decrease starvation risk (Calder, 1974). However, the roles of these genetic and environmental factors are not mutually exclusive (e.g., Barbraud & Jouventin, 1998; Darwin, 1859), and their importance for population divergence still remains to be examined for many species (see Pfennig et al., 2010; West‐Eberhard, 1989), with additional studies from different geographic areas necessary to fully understand mechanisms of morphological variation (Waugh, Prince, & Weimerskirch, 1999; Wojczulanis‐Jakubas et al., 2011).

Yet, to date, the majority of the work on geographic variation in birds has been described for land species, mostly located in temperate regions (reviewed in Ashton, 2002), with very few studies on seabirds (but see Barbraud & Jouventin, 1998; Wojczulanis‐Jakubas et al., 2011; Valenzuela‐Guerra, Morales‐Moraga, González‐Acuña, & Vianna, 2013; Jakubas, Wojczulanis‐Jakubas, & Jensen, 2014). Seabirds are excellent study systems to investigate mechanisms of population divergence. They have the potential to disperse widely and occur over large geographic areas (Marchant & Higgins, 1990), and, as such, gene flow between breeding populations is not restricted by large‐scale physical barriers. Despite this, most populations are geographically isolated as seabirds exhibit high levels of philopatry (where individuals return to their natal colony for breeding; e.g., Reilly & Cullen, 1982; Coulson, 2002; Milot, Weimerskirch, & Bernatchez, 2008) are constraint to forage locally during the breeding season (e.g., Collins, Cullen, & Dann, 1999; Hoskins et al., 2008; Wiebkin, 2012). In addition, due to the higher heat‐absorbing properties of water (compared to air), the Bergmann's rule (larger individuals in colder climates) is predicted to apply even more to diving endotherms (such as seabirds) than to their terrestrial counterparts, and variation in sea surface temperatures has been found as possible drivers for the evolution of body size in kerguelen shags (*Phalacrocorax atriceps verrucosus*) and gentoo penguins (*Pygoscelis papua*) (Bost, Jouventin, & Sel, 1992).

**Figure 1:**
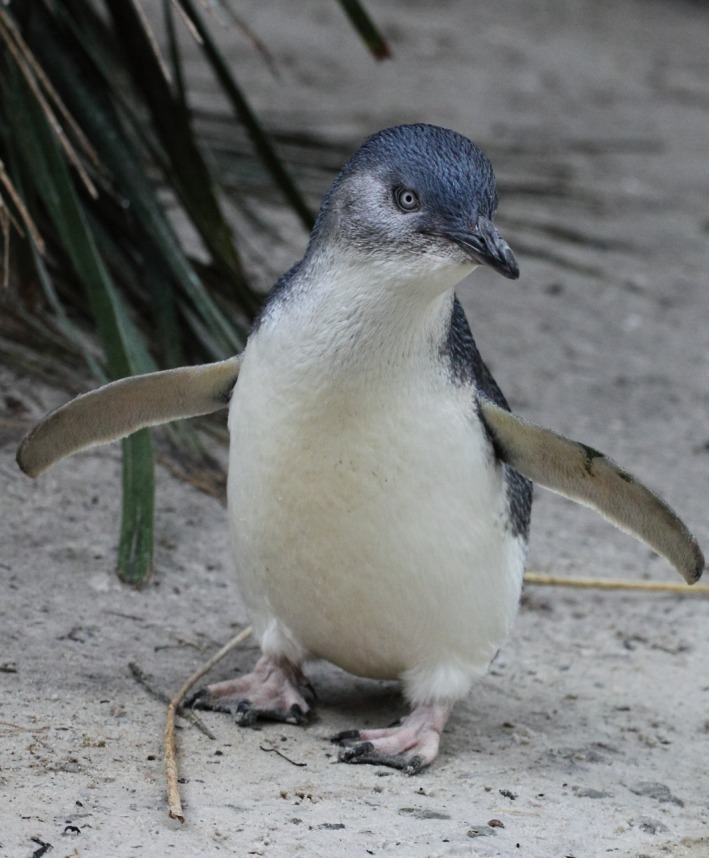
Adult male little penguin (*Eudyptula minor*)

This study focused on morphological variation in little penguins (*Eudyptula minor*; Figure 1), the smallest of all penguin species (Marchant & Higgins, 1990). Little penguins have a large geographic distribution and occupy sites all over the coastlines and offshore islands of South Australia and New Zealand (Marchant & Higgins, 1990). Like most seabird species, they are generally faithful to their breeding site if successful with their first breeding attempt (Bull, 2000; Johannesen, Perriman, & Steen, 2002; Pledger & Bullen, 1998), and most chicks come back to the vicinity of their natal area to attempt to breed as adults (Dann, 1992). Previous studies have shown variation across their range in morphology, diet as well as breeding biology (e.g., Kinsky & Falla, 1976; Klomp & Wooller, 1988; Overeem, Wallis, & Salzman, 2006; Reilly & Cullen, 1981; Wiebkin, 2012). Within Australia, significant differences in bill morphology were found between colonies separated by hundreds and thousands of kilometers (reviewed by Klomp & Wooller, 1988; Arnould, Dann, & Cullen, 2004; Overeem et al., 2006; Wiebkin, 2012). In southeast Australia, Overeem et al. (2006) showed that individuals living east of Cape Otway had significantly smaller bill lengths and depths than those living west. Wiebkin (2012) also showed within South Australia that adults breeding on Troubridge Island had larger bills than those breeding on Pearson Island, supposedly as a result of higher food availability on Troubridge Island. While little penguins are listed as “least concern” nationally (IUCN Red List of Threatened Species, 2014), some local populations have shown a significant decline over the last decades (Colombelli‐Négrel, 2015a; Dann, 1992, 1994; Dann, Norman, Cullen, Neira, & Chiaradia, 2000; Wiebkin, 2011). On Granite Island (South Australia), for example, the little penguin population has fallen from 1,548 individuals in 2001 to only 22 individuals in 2015 (Colombelli‐Négrel, 2016; Wiebkin, 2011). As the reasons for population decline are not fully understood (see Wiebkin, 2011), efforts to gain baseline data and identify variations between populations are important for conservation management.

Here, I investigated small‐scale geographic variation in morphology (bill size and body mass) in little penguins based on samples from five South Australian breeding colonies separated by <150 km. I specifically asked whether adults from different colonies differed in morphology and then investigated whether the differences were related to environmental parameters (air temperature, sea surface temperature, and water depth) or due to isolation by distance. If morphology variation resulted from isolation by distance between the colonies, I expected to find a close relationship between morphology and geographic distances. However, if local adaptation played a role in driving differences in morphology between the colonies, I expected to find some correlation between the environmental parameters and the body size‐related traits. Based on previous studies, I expected that (1) birds breeding in conditions of low air temperatures and/or at sites surrounded by lower sea surface temperatures would be larger than individuals breeding in areas experiencing milder conditions (Bergmann, 1848; Blackburn et al., 1999; James, 1970) and (2) larger individuals would be found at colonies surrounded by shallower waters because they would have access to more accessible prey and consequently have developed larger bodies (Wiebkin, 2012).

## 2 Methods

### 2.1 Study sites

**Figure 2:**
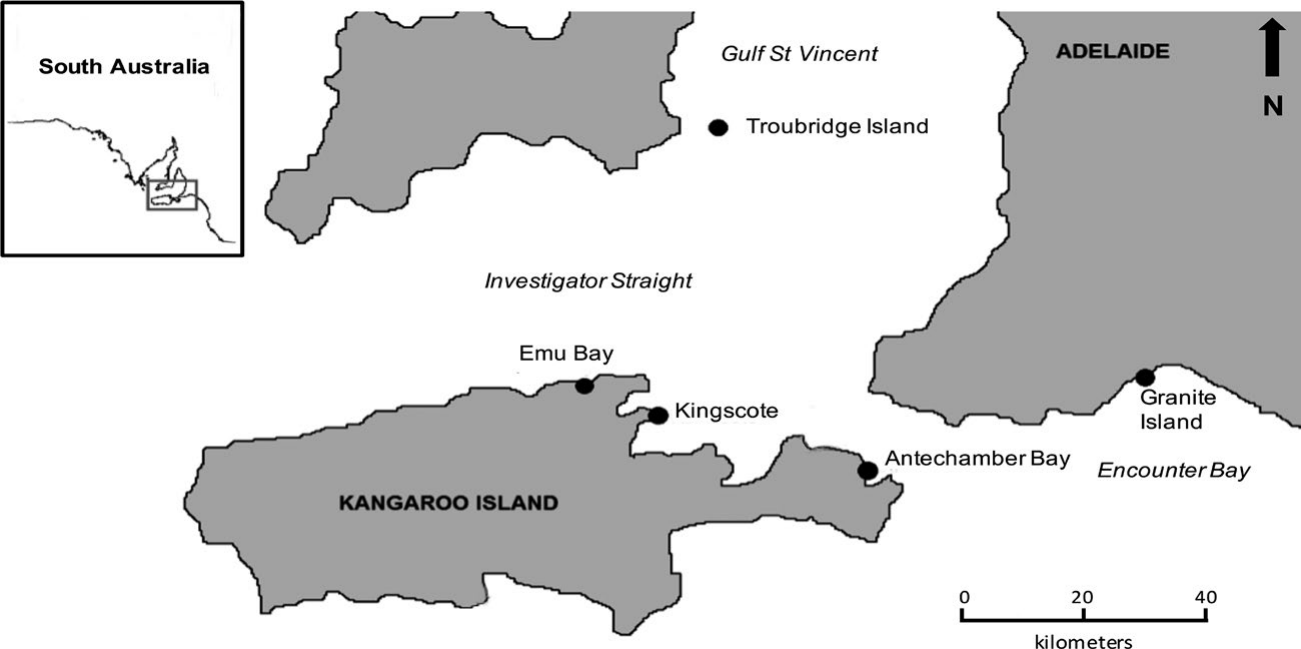
Distribution of the sampled breeding colonies of little penguins (black circles)

Little penguins were morphologically measured between September and January over 3 years (2013/2014–2014/2015–2015/2016) on three islands in the Gulf St Vincent (South Australia): (1) Troubridge Island (35°06′S, 137°49′E)—a sandy island located approximately eight kilometers southeast of Edithburgh (Yorke Peninsula) and mostly dominated with nitre bush (*Nitraria schoberi*) and African boxthorn (*Lycium ferocissimum*); between 300 and 1,000+ adult little penguins were estimated present on this island during each breeding season for the three study years (Bool & Wiebkin, 2013; Colombelli‐Négrel, 2016); (2) Granite Island (35°37′S, 138°36′E)—a small rocky island off Victor Harbor which is connected to the mainland by a bridge causeway and open freely to pedestrians during the day; between 38 and 22 adult little penguins were estimated present on this island during each study years (Colombelli‐Négrel, 2015a,b, 2016; Colombelli‐Négrel & Kleindorfer, 2014); and (3) Kangaroo Island (35°47′S, 137°13′E)—a rocky island 112 km southwest of Adelaide and accessible by ferry. Kangaroo Island is 150 km long and includes several little penguin breeding colonies. Colonies at Antechamber Bay, Emu Bay, and Kingscote were included in this study. Kingscote and Emu Bay are located on the north coast of Kangaroo Island (within approx. 16 km of each other) and Antechamber Bay is located on the eastern side of Kangaroo Island, approx. 50 km away from Kingscote. For the Kingscote colony, little penguins were only monitored along the Hospital Beach, on the northern side of the jetty. All three colonies on Kangaroo Island showed drastic decline during the study years: in 2015, only 10, 42, and 19 adults were estimated present at Antechamber Bay, Emu Bay, and Kingscote (Hospital Beach), respectively (Colombelli‐Négrel, 2016). Each colony was visited every 2 weeks for breeding monitoring as part of another study. Study sites are presented in Figure 2.

### 2.2 Morphology measurements

A total of 105 adults (56 males, 49 females) were captured by hand from their burrow to be morphologically measured with calipers (see Table 1 for specific sample size for each colony). Due to local population declines (Colombelli‐Négrel, 2015b, 2016; Colombelli‐Négrel & Kleindorfer, 2014), sample size in some of the colonies was limited by the number of little penguins present at the time and the practicality for the observer to be able to reach into the burrow to capture the individuals.

**Table 1:** Sample sizes for the little penguin morphological measurements for each colony and sex (male, female)

	Colony				
	Antechamber Bay	Emu Bay	Kingscote	Granite Is.	Troubridge Is.
Male	9	14	4	2	28
Female	2	10	3	5	28
Total	11	24	7	7	56

For all captured individuals, the following measurements were recorded: (1) head length (measured from the back of the head to the tip of the bill); (2) bill length (measured from the tip of the bill to the base of the bill, where the feathers start); (3) bill depth at the base (measured as the vertical thickness of the bill at the base of the bill); (4) bill depth at the nostrils (measured as the vertical thickness of the bill at the nostrils); (5) bill width (measured at the base of the bill); and (6) body mass (weight measured to the nearest 10 g). The majority of measurements were taken by D. Colombelli‐Négrel (*n *=* *74); measurements were also made by S‐L. Reinhold (*n *=* *21) and K. Peters (*n *=* *10). The date of capture and the stage of breeding (not breeding, incubating or with chicks) of each individual were also recorded to test for potential bias in body mass due to breeding activity.

The sex of the individuals was determined using the measurements for bill depth at the nostrils as previously described for little penguins (Arnould et al., 2004; Overeem et al., 2006; Wiebkin, 2012). To ensure that sex was appropriately assessed using morphology measurements, the sex of 46 individuals was also confirmed with a genetic‐based method. To this end, a blood sample (0.01 ml per bird) was collected at the time of capture with a 25‐G needle from the foot vein and stored on FTA paper (Smith & Burgoyne, 2004). Little penguin DNA was extracted from FTA cards using a modified protocol based on Smith and Burgoyne (2004) and sexing protocol followed a modified version of Griffiths, Double, Orr, & Dawson (1998). Specifically, the method used the polymerase chain reaction reagents supplied in the AmpliTaq Gold^®^ 360 DNA Polymerase kit (Thermofisher Scientific) and the primers G1193 and G1194. Polymerase chain reaction was carried out using the Mastercycler^®^ Pro Thermal Cycler (Eppendorf) and the conditions were as follows: 10 min at 94°C; 45× cycle of 45 s at 94°C, 45 s at 48°C, and 45 s at 72°C; 5 min at 72°C; and 2 min at 25°C. Gel electrophoresis was carried out using a 3% agarose gel run at 90 V for 60 min and the 100 bp DNA marker HyperLadder™ IV. Polymerase chain reaction products were stained using GelRed™, and the gel was visualized in the Bio‐Rad Gel Doc™ XR+ System. Two bands and one band (at approximately 300 bp) were indicative of female and male, respectively. The genetic method confirmed that sex was correctly assigned using morphology measurements in 83% of the cases.

### 2.3 Environmental data

Air temperature data (AT, °C) for the last 10 years (2003–2014) were obtained from the Australian Bureau of Meteorology database on the following website http://www.bom.gov.au/climate/data/ using the meteorological stations closest to each breeding colony (distance ranging 0.7–13.6 km; average distance* *=* *6.8 km). Data on sea surface temperatures (SST, °C) within a 20 km radius of ocean surrounding each colony for the last 10 years (2003–2014) were sourced from the Integrated Marine Observing System (IMOS) http://imos.org.au/home.html. Integrated Marine Observing System is supported by the Australian Government through the National Collaborative Research Infrastructure Strategy and the Super Science Initiative. For both AT and SST, only data between August and December were used because they covered the time period when little penguins regularly attend their breeding sites at all of the monitored colonies and a single breeding attempt takes 3 months from egg laying to fledgling. Furthermore, data for the last 10 years (2003–2014), and not just the study years, were used because little penguins are expected to live at least 10 years in the wild (Dann et al., 2005; Nisbet & Dann, 2009), and the age of the individuals was unknown in this study. Mean water depths (WD) within a 20 km radius of ocean surrounding each colony were determined using the 2009 data set available as a 9 arc‐second grid from the Geoscience Australia website: http://www.ga.gov.au (Whiteway, 2009). WD and STT within a 20 km radius were used in this study because they correspond to the foraging range of little penguins during the breeding season (Bool, Page, & Goldsworthy, 2007; Collins et al., 1999; Wiebkin, 2012). The shortest possible distances by sea (distance ranging between 35 and 124 km) between each of the breeding colonies (Troubridge Island, Emu Bay, Kingscote, Antechamber Bay, and Granite Island) were estimated using the measurement tool in Google Earth 7.1 (http://earth.google.com).

### 2.4 Statistical analysis

Statistical analyses were conducted using PASW Statistics software (PASW version 22.0 for Windows, SPSS Inc., Chicago, IL). Data are shown as mean ± SE. The Kolmogorov–Smirnov test showed that all data, except body mass, complied with the conditions of normality. Therefore, body mass was normalized using log_10_ transformation before testing for intergroup differences. Multivariate analysis of variance (MANOVA) was used to compare the morphological measurements taken by the three researchers: the results showed that the measurements were comparable and not statistically different (MANOVA: all *p *>* *.28), and thus, all data were combined. Two analyses of variance (ANOVAs) were used to test for potential bias in body mass due to breeding activity (not breeding, incubating or with chicks) or month of capture (September–January): neither breeding activity (*F*_2, 101_ **=* *0.70, *p *=* *.50) nor month of capture (*F*_3, 101_ **=* *1.10, *p *=* *.35) influenced body mass, and therefore, the data were pooled. Welch ANOVAs (for unequal sample sizes) were first used to assess differences between the colonies and obtain *F*‐ratios. Large *F*‐ratios represent greater between‐ than within‐group variability (Campbell, 1989). Games–Howell post hoc tests (recommended for both unequal sample sizes and variance) were used to identify which of the pairwise comparisons were significantly different. A MANOVA was then conducted to evaluate the influence of the factors “colony” and “sex” on the morphological measurements using the Pillai's trace test statistics as recommended in Quinn & Keough, 2002. Bill measurements were then reduced using principal component analysis (PCA). To examine the relationship between morphology and environmental parameters, linear mixed model analyses were used using sex and colony as fixed factors and the environmental parameters (AT, SST, and WD) as covariates. Estimates of between‐ and within‐group's variance (residual estimates) are presented in the results. Euclidean distances were calculated for all pairwise combinations of colonies using the morphology data. A Mantel test was then used to examine independence between the morphology and geographic distance matrices using XLSTATS version 2015.4.01 (Addinsoft, New York, USA).

## 3 Results

The factor “sex” had the strongest influence on the variability found between the morphological measurements (MANOVA: *F *=* *6.39, *p *<* *.0001). Males were significantly heavier and had significantly greater bill length, bill depth at the base, bill depth at the nostrils, and bill width than females (Table 2). There was no significant interaction effect between sex and colony (all *p *<* *.13), suggesting that males and females differed in the same way across colonies. All morphological measurements, except bill length, showed statistically significant differences between the colonies (Table 2). The largest *F*‐ratios were found for body mass and bill depth at the base (Table 2), indicating that these parameters may be the most important ones used to evaluate geographic differences between the colonies. Mean values as well as the Welch ANOVA and post hoc test (Games–Howell) results for all measurements for each colony are presented in Table 2.

**Table 2:** Mean values ± SE of all the morphological measurements for each colony (1–5) and sex (male, female) as well as the Welch ANOVA results and the Games–Howell post hoc comparisons. The abbreviation for each colony is presented in front of the colony name. In the post hoc results, the first abbreviation represents the colony compared to the other colonies. The following abbreviations are the colonies that are significantly different from this colony. For example, for body mass, there are significant differences between Troubridge Island (TB) and Emu Bay (EB) and between Troubridge Island (TB) and Antechamber Bay (AB) but not between Troubridge Island and the other two colonies (Granite Island and Kingscote)

	*N*	Body mass (g)	Head length (mm)	Bill length (mm)	Bill depth at the base (mm)	Bill depth at the nostrils (mm)	Bill width (mm)
TB. Troubridge Is	53	1,346.0 ± 20.3	103.9 ± 1.0	41.8 ± 0.4	16.5 ± 0.3	13.4 ± 0.2	17.3 ± 0.4
EB. Emu Bay	24	1,197.9 ± 34.8	103.2 ± 1.1	39.8 ± 0.6	16.2 ± 0.4	12.9 ± 0.4	18.8 ± 0.5
K. Kingscote	7	1,221.4 ± 73.9	105.9 ± 0.6	40.7 ± 0.7	17.3 ± 0.5	13.6 ± 0.4	19.7 ± 0.7
AB. Antechamber Bay	11	1,139.1 ± 31.8	99.8 ± 1.3	41.1 ± 0.9	17.9 ± 0.4	14.5 ± 0.4	16.8 ± 0.6
GI. Granite Is	7	1,100.0 ± 68.1	98.2 ± 1.5	40.2 ± 0.9	21.0 ± 1.3	14.1 ± 0.4	17.4 ± 0.5
Welch ANOVA		*F *=* *9.12*p *<* *.0001	*F *=* *2.50*p *<* *.0001	*F *=* *2.14*p *=* *.12	*F *=* *8.79*p *=* *.007	*F *=* *2.38*p *=* *.05	*F *=* *2.46*p *=* *.02
Post hoc tests showing significant differences		TB–AB/EB	K–AB/GITB–GI		TB–EB	AB–EB	AB–K
Males	57	1,308.2 ± 21.6	104.6 ± 0.7	41.9 ± 0.3	17.7 ± 0.3	14.2 ± 0.2	18.7 ± 0.3
Females	45	1,206.7 ± 26.8	101.2 ± 1.0	40.2 ± 0.5	15.9 ± 0.3	12.6 ± 0.2	16.7 ± 0.4
Effect of “sex” (main‐effect MANOVA)		*F *=* *11.03*p *=* *.001	*F *=* *2.27*p *=* *.13	*F *=* *11.91*p *=* *.001	*F *=* *18.45*p *<* *.0001	*F *=* *16.12*p *<* *.0001	*F *=* *7.31*p *=* *.008

**Figure 3:**
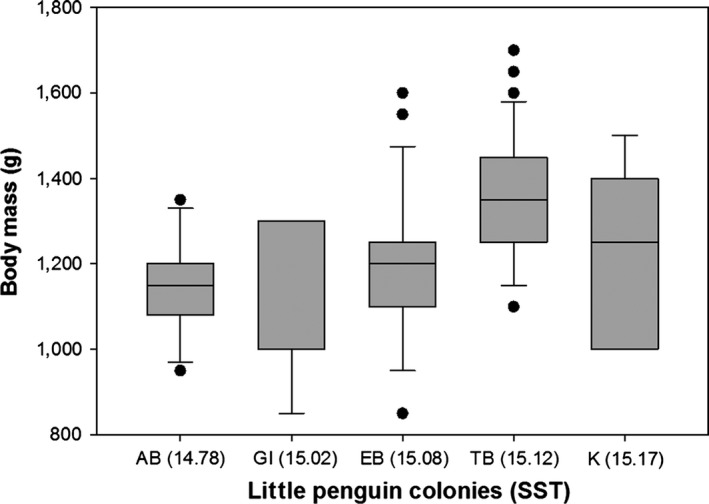
Little penguin body mass (g) (±SE) in relation to mean sea surface temperatures (SST; °C) and penguin colonies (Troubridge Island, TI; Emu Bay, EB; Kingscote, K; Antechamber Bay, AB; Granite Island, GI): Birds breeding at colonies surrounded by hotter sea surface temperatures were heavier than those breeding at colonies surrounded by cooler waters

**Figure 4:**
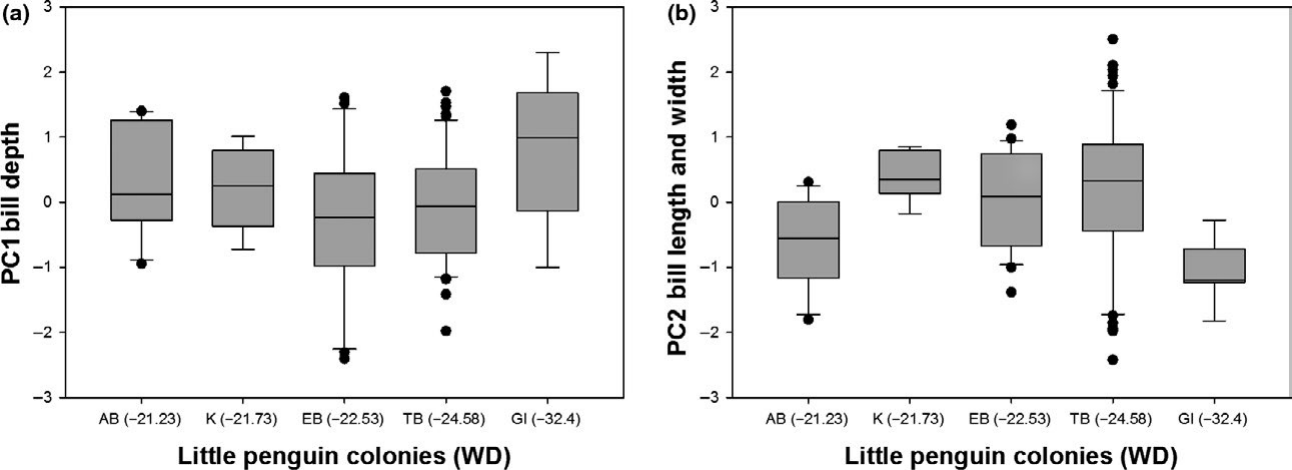
Little penguin bill parameters (shown as PCA factor scores ± SE) in relation to the depth of the water surrounding the colonies (WD; m) and the colonies (Troubridge Island, TI; Emu Bay, EB; Kingscote, K; Antechamber Bay, AB; Granite Island, GI): Birds with larger bill depths and widths and longer bills were found at colonies surrounded by shallower waters. The data are presented for (a) PC1 bill depth and (b) PC2 bill length and width

**Figure 5:**
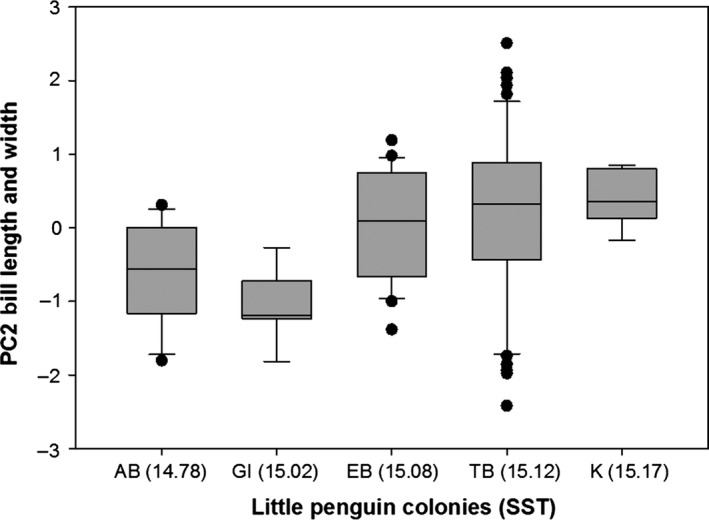
Little penguin bill length and width (shown as PCA factor scores ± SE) in relation to mean sea surface temperatures (SST; °C) and penguin colonies (Troubridge Island, TI; Emu Bay, EB; Kingscote, K; Antechamber Bay, AB; Granite Island, GI): Birds at colonies surrounded by hotter sea surface temperatures had longer bills and larger bill widths

**Figure 6:**
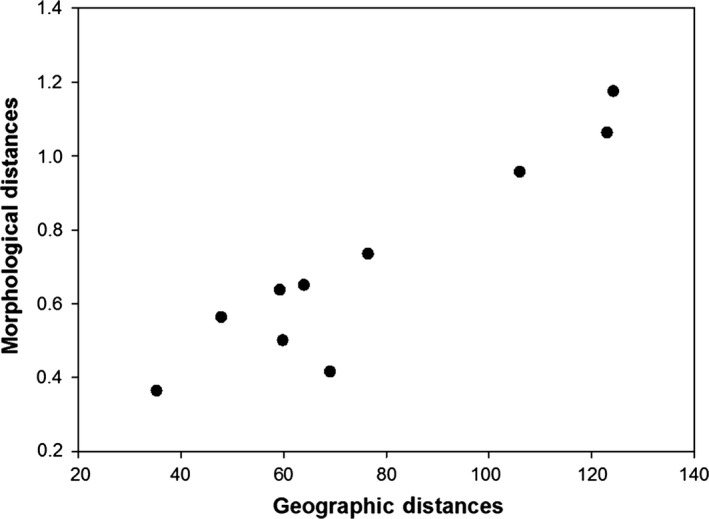
Results of the matrix of geographic distances (km) between the colonies in relation to the matrix of differences in morphology

Variation in body mass was associated with variation in AT (*F *=* *15.25, *p *<* *.0001) and STT (*F *=* *37.68, *p *<* *.0001; Figure 3) and differed between sexes (*F *=* *16.01, *p *<* *.0001) but was not associated with variation in WD (*F *=* *0.11, *p *=* *.74). Specifically, heavier birds were found at colonies with hotter sea surface temperatures and cooler air temperatures (residual variance estimate* *=* *0.002). PCA bill provided two components with eigenvalues >1, which explained 66% of the variance: 37% of the variance was accounted for by PC1 bill depth (high factor loading for bill depth at the nostrils and bill depth at the base) and 29% was accounted for by PC2 bill length and width (high factor loading for head length, bill length, and bill width) (Table 3). Variation in PC1 bill depth was associated with variation in WD (*F *=* *17.627, *p *<* *.0001; Figure 4a) and differed between sexes (*F *=* *59.80, *p *<* *.0001) but was not associated with variation in AT (*F *=* *0.000, *p *=* *.98) or SST (*F *=* *1.52, *p *=* *.22). Birds with larger bill depths were found at colonies surrounded by shallower waters (residual variance estimate = 0.59). Variation in PC2 bill length and width was associated with variation in WD (*F *=* *5.20, *p *=* *.02; Figure 4b) and SST (*F *=* *5.04, *p *=* *.03; Figure 5) and differed between sexes (*F *=* *6.53, *p *=* *.01) but was not associated with variation in AT (*F *=* *0.05, *p *=* *.82). Birds with longer bills and larger widths were found at colonies surrounded by hotter sea surface temperatures and deeper waters (residual variance estimate 0.84). Estimates of between‐group variance are summarized in Table 4. A significant correlation was also found between the geographic distances between the colonies and differences in the morphological parameters (Mantel test: r = .94, *p *=* *.001; Figure 6).

**Table 3:** Factor loadings from principal component analysis (PCA) of the bill measurements. High PCA scores indicate larger measurements

	PC1 bill depth	PC2 bill length and width
Head length	0.076	0.857
Bill length	0.457	0.583
Bill depth at the base	0.864	−0.328
Bill depth at the nostrils	0.888	−0.213
Bill width	0.311	0.452

**Table 4:** Factors driving the geographic variation in morphology of the little penguins in South Australia. Estimates (between‐group variance; ±SE) from the mixed models analysis are presented for body mass and the two PCA bill components in relation to the sex of the individuals (male, female) and the environmental parameters (AT, air temperature; STT, sea surface temperature; and WD, water depth)

Variables	Estimate	SE	*t*	*p*
Body mass				
Sex	0.04	0.01	4.01	<.0001
AT	−0.05	0.01	−3.90	<.0001
WD	<0.001	0.02	0.34	.74
STT	0.49	0.08	6.14	<.0001
PC1 bill depth				
Sex	1.20	0.15	7.73	<.0001
AT	−0.003	0.18	−0.02	.98
WD	−0.13	0.03	−4.20	<.0001
STT	−1.51	1.22	−1.23	.22
PC2 bill length and width				
Sex	0.47	0.18	2.56	.01
AT	−0.05	0.22	−0.23	.82
WD	0.08	0.04	2.24	.03
STT	3.32	1.46	2.28	.02

## 4 Discussion

In this study, I showed substantial morphological variation in body mass and bill measurements (except bill length) among five South Australian little penguin colonies separated by <150 km. Phenotypic trait variation were correlated to environmental parameters but were also influenced by interpopulation distances. To the best of my knowledge, this is the first evidence for the combined roles of both genetic and environmental factors on population divergence in a seabird species. Specifically, I found that individuals breeding in colonies further located from each other showed greater overall morphological divergence than those breeding in adjacent colonies. In addition, the morphological traits were somewhat correlated to local environmental parameters: (1) little penguins breeding in cooler air temperature conditions were heavier than those breeding in hotter conditions; (2) birds at colonies surrounded by hotter sea surface temperatures were heavier with longer bills and larger bill widths; and (3) birds with larger bill depths and widths and longer bills were found at colonies surrounded by shallower waters.

Many seabird species show high levels of natal and breeding philopatry (Coulson, 2002; Milot et al., 2008; Reilly & Cullen, 1982), thus making breeding populations very isolated geographically with little or no gene flow (reviewed in Friesen, 2015). Despite this, most studies on seabirds to date found no correlation between body traits and geographic distances (Waugh et al., 1999; Bull, 2006; Peck, 2006; Wojczulanis‐Jakubas et al., 2011; but see Power & Ainley, 1986). Recent molecular analyses between the same little penguin colonies studied here showed a pattern consistent with isolation by distance, where pairwise coefficients of relatedness decreased with distance (Colombelli‐Négrel, 2015b; Graff, 2015). But in contrast to other studies on seabirds, this study found a significant correlation between overall divergence in morphology and the geographic distances, with individuals from adjacent colonies being morphologically more similar than those located in colonies further apart from each other. While little penguins are mainly philopatric, there is evidence of individuals breeding at locations other than their natal colony (Dann, 1992; Priddel, Carlile, & Wheeler, 2008; Reilly & Cullen, 1982). In this study, morphological similarity between the two closest colonies (Emu Bay and Kingscote on Kangaroo Island) is therefore likely to be the result of gene flow between the colonies and similarity in environmental parameters as the foraging zones of the individuals breeding at those two colonies are likely to overlap. The lack of relationship between body traits and geographic distances observed in other seabird species could be due to variation in levels of philopatry across species. In addition, all studies so far investigating morphological variation in relation to environmental parameters and geographic distances in seabirds have focused on species with the ability to fly, while this is the first study investigating this question in a flightless species. Additional studies with different species are clearly needed to elucidate the potential impact(s) of genetic factors on population differentiation in seabirds.

Individuals living in colder climates are generally larger in body size or mass, allowing them to store larger amounts of body reserves to better preserve heat (Bergmann, 1848; Blackburn et al., 1999; James, 1970; Symonds & Tattersall, 2010) or decrease starvation risk (Calder, 1974). In support for this idea, I found that little penguins breeding in cooler air temperature conditions were heavier than those breeding in hotter conditions. However, the between‐group estimate of variance was very small (−0.05) and lower than the between‐group estimate of variance for the relationship between body mass and sea surface temperature (0.49; Table 4). Indeed, I also found that little penguin body mass increased with increasing sea surface temperature. Specifically, individuals breeding on Troubridge Island and at the Kingscote colony (where the hotter sea surface temperatures were found) were the heaviest, while individuals from Granite Island and Antechamber Bay (where the cooler sea surface temperatures were found) were the lightest. For diving endotherms, such as little penguins, it is also more likely that individuals would respond more to changes in sea surface temperatures rather than air temperatures and hence the relationship between body mass and air temperature may not have any biological relevance. Lindstedt and Boyce (1985) have shown that larger body size may actually be more favorable in more seasonal environments because larger animals can store more fat for stressful times. This would be particularly applicable for little penguins as carryover body mass has been shown to increase their chances of survival (Dann, 1988; Reilly & Cullen, 1982) and improve reproductive success (Salton, Saraux, Dann, & Chiaradia, 2015). However, considering the relatively low variation in sea surface temperatures observed here (mean ranging from 14.78°C to 15.17°C), the correlation with the sea surface temperature should also be taken with caution. In addition, it is possible that other differences between the colonies (such as variation in prey availability, species, or catch depth; see Cook et al., 2013) may be driving the differences in body mass. In support for this hypothesis, work by Wiebkin (2012) on fledgling growth from Troubridge and Pearson Islands showed that higher food availability on Troubridge Island led to larger fledglings and consequently larger adults. Differences in body mass also reflect differences in energy reserve as well as interindividual differences in structural body size. Calculations of body condition indexes (body mass corrected for body size, thus reflecting energy reserves; Peig & Green, 2009) showed exactly the same trends as those observed for body masses (data available upon request). This therefore suggests that differences in body reserve—rather than body size—may be driving the colony differences observed here.

In order to reduce thermoregulatory costs, the Allen's rule (Allen, 1907) hypothesizes that endothermic species should have smaller extremities (such as tails or limbs) in colder environments. In birds, there is evidence that bill size and shape can play an important role for heat exchange (e.g., Greenberg, Cadena, Danner, & Tattersall, 2012; Hagan & Heath, 1980; Symonds & Tattersall, 2010; Tattersall, Andrade, & Abe, 2009), with up to 60% of the body heat lost through the bill (Tattersall et al., 2009). In accordance with Allen's rule, I found that bill length and width (PC2) increased with increasing sea surface temperature. However, bill size may also be under other selections such as for foraging, and bill size and shape have been found to vary in relation to foraging and/or intraspecific competition in other seabird species (Kohler et al., 2014; Mancini, Hobson, & Bugoni, 2014). For example, in some populations of African black oystercatchers (*Haematopus moquini*), females feed more on mussels and have longer and more pointed bills while males focused more on limpets and have blunted bills (Kohler et al., 2014). In penguins, larger bills have also been shown to increase the efficiency of obtaining food (Agnew & Kerry, 1995; Wiebkin, 2012). In support for the hypothesis that multiple selections act on bill parameters, bill length and width (PC2) also varied with water depth, which would be important for foraging. Therefore, this study highlights the importance of investigating more than one parameter to fully understand correlations between morphology and environmental variables.

Diving in deep waters is considered a costly behavior because the time for recovery increases more with water depth than with diving time (Kooyman & Ponganis, 1998). Therefore, individuals are expected to develop an optimal body size for deep diving and individuals that regularly need to forage in deeper waters may benefit from being larger (Cook et al., 2013). In contrast, I found here that birds with larger bill depths (PC1) were found at colonies surrounded by shallower waters than birds with smaller depths. However, my results align with results from Wiebkin (2012) who found a negative correlation between little penguin bill depth at the nostrils and the depth of the waters surrounding the colonies. The author also found a positive correlation between bill depth at the nostrils and overall body size (with larger individuals having larger bill depth) and suggested that regular diving in shallower waters may have provided individuals with more prey capture opportunities (see also Ropert‐Coudert, Kato, Wilson, & Cannell, 2006), which in turn may have increased chick growth (including bill) leading to larger adults in her study (Wiebkin, 2012). While I did not test statistically for a relationship between bill depth and overall body size in my study, I do not except to find the same relationship. In the present study, the largest individuals (in terms of body mass) were found on Troubridge Island but the individuals with the largest bill depth at the base were found on Antechamber Bay. Regardless of the overall size of the individuals, it is still possible that a similar pattern to the one suggested by Wiebkin (2012) exists, with individuals breeding at colonies surrounded by shallower waters having better access to more accessible prey, which may have resulted in development of larger bill depth at the base. It should also be noted that the between‐group estimate of variance for bill depth (PC1) was higher for the sex difference (Table 4), thus suggesting that bill depth may also be under multiple selections.

This study found significant morphological variation between colonies separated by <150 km, which aligns with other studies on little penguins showing differences in bill morphology between colonies separated by few hundred kilometers (Arnould et al., 2004; Wiebkin, 2012). As found by Wiebkin (2012), individuals on Troubridge Island were the largest for most of the morphological parameters measured (Table 2), which may be due to the presence of more accessible prey as suggested by Wiebkin (2012). Klomp and Wooller (1988) also suggested that more favorable food supply could explain why heavier individuals with longer bills were found on Penguin Island (Western Australia) compared to those elsewhere in Australia. While little penguins were originally divided into six discrete subspecies (five in New Zealand and one in Australia) based on morphometric measurements and plumage coloration (Kinsky & Falla, 1976), recent genetic analyses found only two clades in Australasia (Banks, Cruickshank, Drayton, & Paterson, 2008; Banks, Mitchell, Waas, & Paterson, 2002; Peucker, Dann, & Burridge, 2009). Genetic studies within the southeast Australia showed some genetic differentiation for the populations located in South Australia (Burridge, Peucker, Valautham, Styan, & Dann, 2015; Graff, 2015; Overeem, Peucker, Austin, Dann, & Burridge, 2008), but only Troubridge Island differed genetically from all the other sampled colonies in this study (Colombelli‐Négrel, 2015b; Graff, 2015). Indeed, a recent genetic study of the South Australian colonies identified two genetically distinct populations: the first population included Emu Bay, Kingscote, Penneshaw, Antechamber Bay, and Vivonne Bay (all on Kangaroo Island), as well as Granite and Althorpe Islands, and the second population consisted solely of Troubridge Island (Colombelli‐Négrel, 2015b; Graff, 2015). This, therefore, suggests that differences in morphology are not represented by differences in genetics, as documented in other seabird species (Genovart, Oro, & Bonhomme, 2003; Moen, 1991; Randi, Spina, & Massa, 1989). This may also raise questions as to whether additional management measures may be needed to preserve some of these populations. As mentioned before, the population on Granite Island has fallen from 1,548 individuals in 2001 to only 22 individuals in 2015 (Colombelli‐Négrel, 2016). Similarly, most populations on Kangaroo Island—which were considered unsure or stable until now—are showing similar trends with more than 50% of local decline (Natural Resources Kangaroo Island, 2014). While the genetic results suggest that the loss of local populations (except for Troubridge Island) should not result in major losses of genetic variability, additional studies investigating the importance of population differentiation for the species (as a unit) across more colonies, both within South Australia and within the whole range, may be needed to ensure that distinct (and maybe relevant) morphological, behavioral, and ecological traits are (or continue to be) preserved.

## Conflict of Interest

None declared.
